# Israeli Position Paper: Triage Decisions for Severely Ill Patients During the COVID-19 Pandemic. Joint Commission of the Israel National Bioethics Council, the Ethics Bureau of the Israel Medical Association and Representatives from the Israeli Ministry of Health*

**DOI:** 10.5041/RMMJ.10411

**Published:** 2020-07-31

**Authors:** Avraham Steinberg, Ephrat Levy-Lahad, Tami Karni, Noam Zohar, Gil Siegal, Charles L. Sprung, the Israel National Bioethics Council, the Ethics Bureau of the Israel Medical Association, and Representatives from the Israeli Ministry of Health

**Affiliations:** 1 Medical Ethics Unit, Shaare Zedek Medical Center, Jerusalem, Israel; 2 Co-Chairman, Israel National Bioethics Council, Jerusalem, Israel; 3 Medical Genetics Institute, Shaare Zedek Medical Center, Jerusalem, Israel; 4 Faculty of Medicine, Hebrew University of Jerusalem, Jerusalem, Israel; 5 Head, Breast Care Institute, Assaf Harofe Medical Center, Zerifin, Israel; 6 Chairman, Ethics Bureau of the Israel Medical Association, Ramat Gan, Israel; 7 Head, Advanced Studies in Bioethics, Faculty of Philosophy, Bar-Ilan University, Ramat Gan, Israel; 8 Director, Center for Health Law & Bioethics, Faculty of Law, Ono Academic College, Kiryat Ono, Israel; 9 Department of Anesthesiology, Critical Care Medicine and Pain, Hadassah Medical Center, Jerusalem, Israel

**Keywords:** COVID-19 pandemic, intensive care, national policy, triage, ventilators

## Abstract

**Objectives:**

This document provides an English translation of the Israeli Joint Commission’s national guidelines for triaging severely ill patients during the coronavirus disease 2019 (COVID-19) pandemic.

**Methods:**

Four subcommittees of medical, legal, ethical-social, and religious experts developed the general principles and practical medical criteria for triaging scarce life-saving resources.

**Results:**

The guidelines provide an overview of general principles as well as pragmatic medical criteria and a practical triage protocol to be followed should the healthcare system be overwhelmed due to COVID-19. Issues covered include triggers for activating the guidelines, guiding ethical, legal, and religious principles, equity in access, fair distribution, transparency, consistency, palliation, medical policy prioritization, problem-solving mechanisms, and public trust.

**Conclusions:**

The Israeli consensus document and pragmatic medical triage protocol offer a societal and medical roadmap for allocating scarce resources during the COVID-19 pandemic or other disasters.

## Chapter 1: Introduction and Purpose

### I. Introduction

The global Coronavirus pandemic has created a severe, difficult and tragic ethical-social-religious-legal dilemma because of the need to make triage decisions for the treatment of severely ill patients requiring ventilation and intensive care. The significance of these tragic decisions is that in the absence of sufficient life-saving resources and skilled staff to simultaneously treat all patients requiring life-saving treatments some severely ill patients will not receive optimal life-saving treatment. Therefore, these are real and tangible questions of immediate life and death.

Coronavirus infections (SARS-CoV-2) can be asymptomatic or may vary in severity, ranging from mild flu-like symptoms and varying degrees of difficulty in breathing to interstitial pneumonia accompanied by severe hypoxemia. The severely ill require hospitalization in intensive care units (ICUs) and the most severely ill patients need ventilation using respirators and sometimes extracorporeal membrane oxygenation (ECMO; a type of heart-lung machine). In view of the large number of infected people, especially in view of the large number of patients who will concurrently require intensive care and ventilators for prolonged periods of time, several countries have had insufficient health resources and have had to triage the provision of complex respiratory care. These countries differ in their definitions for the criteria for triage.

The issue of establishing criteria for triaging scarce life-saving resources and skilled manpower has long been known in relation to various forms and causes: situations that are defined as multi-casualty events such as motor vehicle accidents with many injured; natural disasters such as earthquakes, floods, tornadoes, tsunamis, etc.; wars with many injured from conventional and unconventional weapons; industrial disasters such as fires, explosions, toxic leaks etc. Pertinent to the current situation: epidemics of the plague, cholera, influenza, Ebola, AIDS, etc.

These commonly involve a sudden and unexpected event in which many people are injured to varying degrees within a short period of time and when there is limited capacity of the medical system to provide complete solutions for all the casualties because of the lack of manpower and sufficient resources and due to the required speed for interventions.

A country can decide that medical teams will have the freedom of flexible conduct in emergencies without publicly-set normative requirements. This Commission, however, believes that there is a clear preference for advance societal determination of ethical-legal-religious norms and of the methods for their medical implementation, so as to facilitate uniform and transparent professional medical practice. There is no intention, nor is there a realistic ability to dictate in advance the conduct for every situation and case as there are always borderline, delicate and complex cases that require specific attention. But in most cases, triage decisions can and must proceed in a structured and transparent manner according to clear ethical, legal, social and religious standards. Proceeding in such a manner can best ensure equality of rights between patients, while also enhancing public trust in the medical professionals who unquestionably work well beyond the call of duty for the sake of their patients, sometimes even at their own risk.

It should be emphasized that the current situation in the State of Israel – at the time of publication of this position paper – is not one of scarcity of resources or manpower for treating patients requiring intensive care or ventilation. Therefore, in the present situation, it is mandatory to provide equal treatment to everyone without restrictive criteria, following the same practices as in routine situations. In particular it should be noted that neither in the current situation nor in a situation of scarce life-saving resources should there be any treatment preference either favoring or disfavoring non-COVID-19 patients versus COVID-19 patients.

*The current situation where treatment is provided to everyone in need without restrictions must continue as long as the Ministry of Health has not declared that the health care system has reached insufficiency of resources. It is then and only then that the medical, ethical, legal and religious rules set out in this position paper shall apply*.

This position paper is thus intended to provide medical, ethical, social, legal and religious responses if and when there arises a significant shortage of resources and manpower to treat all patients who require intensive care and ventilation, whether in the ICU or in a regular ward, producing a need to triage among patients. Such triage decisions involve as noted difficult decisions, but it is important to understand that the very need for intensive care and ventilation is indicative of the severity of the patient’s medical condition. The current information on COVID-19 patients shows that, unfortunately, most of those in need of ventilation are not likely to recover even if provided with mechanical ventilation.

*The mandate* given to this Commission by the Ministry of Health is to discuss the issue of allocation of life-saving measures and skilled personnel in the event of scarcity of these resources. Therefore, the position paper only addresses this question in its broader context, but not the full range of issues arising from the emergency of the COVID-19 pandemic.

### II. Purpose

The purpose of this position paper is twofold: a) to establish ethical, social, legal and religious guiding principles; and b) to determine the applicable medical-therapeutic considerations and criteria in a medical emergency during the COVID-19 pandemic – both when there is no shortage of life-saving resources and skilled manpower and when such a shortage exists.

These principles come to preserve a degree of humanity, compassion, justice and love for others even in a time of existential crisis; to assist the medical team in making the most just and transparent decisions in triaging severely ill patients for admission to the ICU or for ventilation, whether in the ICU or in a regular ward; to relieve the heavy emotional burden that may be borne by the medical staff facing this difficult dilemma; and to help the public cope with an extreme condition of having to triage treatment in order to save the most patients while applying principles of justice and equality.

### III. Establishment of the Commission and Its Composition

This public Commission was established on April 2, 2020 following its appointment by the Director General of the Ministry of Health on April 1, 2020. The Commission combines the Israel National Bioethics Council, the Ethics Bureau of the Israel Medical Association and the Israeli Ministry of Health. This is a multidisciplinary Commission, represented by senior experts in the fields of medicine, ethics, law, society, Jewish law, Christianity and Islam. The Commission consists of 26 members, a coordinator, research assistant and secretary. The members of the Commission represent the following areas: medicine, ethics, philosophy, sociology, social work, law, halacha (Jewish religious law), Islam and Christianity. In order to streamline the work on the one hand and to exploit the relevant multidisciplinary aspects on the other, the Commission was divided into 4 subcommittees: medical, philosophical-ethical-social, legal, and religious – Jewish, Islam and Christianity. Appendix 1 lists the members of the Commission and of the subcommittees.

The subcommittees and the plenary Commission held regular meetings (via email and video calls due to the ban on congregating) and reached unanimous agreement without reservations on the relevant principles in ethics, law, and religion and on the practical medical guidelines and criteria pertaining to triaging seriously ill patients who require mechanical ventilation and ICU admission. The Commission made a considerable effort in a short period of time to encompass the full range of relevant aspects of this difficult issue and to present the issues clearly and transparently.

## Chapter 2: Ethical, Legal and Religious Principles

### 1. Principles of the Value of Life and Equality in Preserving Human Life

1. The principal starting points for treating patients in need of medical resources are the supreme value of life and the basic equality of all human beings.
2. “Therefore Adam was created alone, to teach you that whoever destroys a single life is deemed by Scripture as if he had destroyed a whole world; and whoever saves a single life is deemed by Scripture as if he had saved a whole world.”^1^ The value of each and every life is paramount and hence there is a moral, legal and religious duty to do as much as possible to save the life of every person.
3. This concept is grounded in the State of Israel’s Basic Law: Human Dignity and Liberty, and in various Supreme Court rulings which include the rights to life, health, dignity and equality. In addition, there are laws that stipulate various arrangements for maintaining this principle, such as the Patient Rights Law 1996, the National Health Insurance Law 1994, the Public Health Ordinance 1940, the Equal Rights for People with Disabilities Law 1998, and the Dying Patient Law 2005.
4. There is a presumption that every person wants to continue living, unless he is a dying patient, defined as a patient whose life expectancy is no longer than six months even with medical treatment, and it is proven beyond reasonable doubt that he does not want his life prolonged.
5. The ethical and legal requirement of equality prohibits any discrimination between patients for religious, race, gender, nationality, country of origin, sexual orientation, socioeconomic status, social status, marital status, citizenship, occupation, disability, age, etc.

### 2. Duties of the State and Citizens to Uphold the Values of Life and Equality

6. During normal times and during an emergency when there is no shortage of life-saving resources and skilled manpower, the state must do all that is necessary and possible – while maintaining proper and proportionate balance with other vital public needs – to provide all that is necessary to preserve as much as possible the supreme value of life and the value of equality. It will thus prevent or postpone as much as possible a situation requiring tragic and fateful decisions of triage decisions in treatment.
7. Indeed, in the current situation (while this position paper is being published) the State of Israel is making tremendous efforts to quickly build temporary ICUs, acquire and manufacture ventilators, procure and manufacture personal protective equipment for healthcare teams, and train appropriate medical staff to treat ventilated patients.
8. The State must also make efforts to allocate the means for providing palliative care including skilled multidisciplinary staff, pharmaceuticals and equipment.
9. Resources should be directed to COVID-19 patients, including space and personnel, provided that this does not adversely affect the care required for non-COVID-19 patients with the same degree of severity. For example, hospitals should utilize specially adapted areas for ICU services such as procedure areas, recovery rooms, operating rooms, and step-down units in order to receive ventilated patients – COVID-19 and non-COVID-19 patients – at the same time redirecting personnel from routine tasks to treating pandemic patients. Yet, the number of beds or medical staff in other wards should not be reduced in a way that would increase risk to their patients to a degree exceeding the reduction in risk to COVID-19 patients.
10. The State must ensure the nationwide fair distribution of available ICU beds, ventilators and skilled personnel required to operate them according to population density and expected/actual demand for these scarce resources. To this end, the ratio of the number of inpatients in the COVID-19 wards to the number of intensive care units in each medical center should be monitored centrally and the hospitalization of the patients should be adjusted to maintain an equal ratio as far as possible. If in some areas of the country the gap between needs and resources results in unsatisfactory treatment, patients should be transferred to less busy hospitals in order to provide comparable treatment in all areas of the country.
11. Responsibility at the national level must also include responsibility for persons who do not have the status of either citizens or residents of the state.
12. The general moral responsibility of the State of Israel must be recognized regarding the health of the Palestinians in the territories under full or partial control of Israel, as well as its duty to assist the Palestinian Authority in dealing with the danger of the pandemic. The extent of assistance will be determined by the State of Israel authorities.
13. As a general rule nursing institutions or mental health departments should not be closed or evacuated to prepare inpatient wards for COVID-19 patients. Transfer of patients from such institutions and departments “to the community” or to their homes often returns them to grievous situations of neglect or abuse. At the same time, at an early stage of the pandemic plans should be made for the release of patients not requiring hospitalization and for restricting the admission of patients for whom there is no urgency of hospitalization, in order to increase the number of beds available.
14. The Commission recommends that the Ministry of Health explore appropriate ways for legal protection of medical staff, who work in difficult, high-risk conditions and must make difficult and quick decisions under the pandemic’s unique conditions.
15. The Commission recommends that the Ministry of Health explore the need to introduce regulations or legislative amendments as required by the guidelines in this position paper.
16. There is a duty for every citizen in the country to strictly adhere to all government guidelines to prevent becoming infected and infecting others, so as to prevent severe morbidity that could lead to overwhelming the health care system.

### 3. Partial Inequality in Situations of Emergency and Scarcities

#### 3A. Principles for determining triage decisions

17. In an emergency, if and when there are many patients in numbers exceeding the resources and manpower that can provide optimal medical care for each patient, then ethical and religious considerations change compared to the normal situation and the ethical and religious focus shifts to the commitment to the health and lives of the public as a whole, sometimes at the expense of individuals.
18. In such a situation, changes and adjustments in triage considerations that affect the standard of treatment are required, depending on the actual situation.
19. The principal rule for triaging patients in situations of emergency situations and resource scarcity is to save as many people as possible, according to the principle of providing the greatest good to the greatest number. The only deviation from the principle of equality is on the basis of medical considerations for the success of the treatment and the chances of survival and weaning from the respirator or ECMO.
20. Therefore, even in emergencies and shortages, there must be no discrimination among patients based on non-medical characteristics or on the general characteristics of groups of people. Individual triage should be determined according to the specific medical data for each patient.
21. The legal-ethical principle in these cases is the principle of proportionality and the balance between various considerations, according to which the necessary deviation from the principle of equality is considered in a structured, transparent and fair manner.
22. Therefore, if there are patients whose severe medical condition requires life-saving treatment without which they will die on the one hand, and their medical chances of benefiting from the life-saving treatment promise greater chances of survival on the other hand, they have precedence over patients who can survive without life-saving treatments and over patients whose medical prospects, even if provided with life-saving treatment, are slim.
23. To that end, there are medical assessment systems that physicians can employ to assess the likelihood that these treatments will be needed and the likelihood of survival of patients receiving these treatments.

#### 3B. Principles for implementing triage

24. The principles of triage decisions apply to all treatment modalities in short supply – hospitalization in intensive care versus in a regular ward, use or non-use of ventilators or ECMO, etc.
25. During a pandemic, there is a need to restrict intensive care admission to either achieving benefit or improvement in a limited period of time, facilitating discharge of patients to a non-ICU ward, or conversely to limit the intensity of treatment provided in the event of deterioration of the medical condition.
26. Therefore, patients who have been admitted to the ICU, whether breathing spontaneously or on a ventilator, and whose condition has deteriorated so that their chance of survival is very low, should be considered for discharge from the ICU and transfer to a regular ward in order to treat other patients whose chances of survival are greater.
27. In addition, ventilated patients whose condition deteriorated and whose chances of survival are poor, consideration should be given to refraining from further intermittent life-saving treatments, such as dialysis, vasopressor agents, etc.
28. The Commission discussed at length the question of discontinuing a patient from a ventilator if his chances of survival are slim. This question is very sensitive and highly contested in Israeli society; it was likewise contested among the Commission members with opinions roughly equal. The Commission believes that at this stage no decision is required on this question, since the actual situation in Israel at the time of this position paper certainly does not require such a decision. Even if we do reach a situation where there is a shortage of life saving resources and manpower, the Commission believes that this position paper contains sufficient triage mechanisms that should not require the discontinuation of ventilators. Moreover, it appears that withdrawing a ventilator from a patient is currently illegal in the State of Israel (according to paragraph 21 of the Dying Patient Act). If and when it becomes clear that the question is real, the Commission will convene for a swift discussion and decide.

#### 3C. The patient’s will

29. Ethically, one should ascertain the patient’s desire for invasive treatments in general and mechanical ventilation in particular. This approach is also reflected in the requirement of obtaining consent prior to performing any medical procedure and in the Dying Patient Act, which allows the provision of advance medical directives and the appointment of a durable power of attorney for end-of-life medical decisions. Despite the long time since that law was enacted, to date only a small minority of Israelis have written advance medical directives or appointed a durable power of attorney for medical decisions.
30. It should be noted that relatives who are not the patient’s legal guardians or appointed by a durable power of attorney have no special status regarding decisions for a patient who cannot express his will. However, in the case of a patient who neither wrote an advance medical directive nor recorded a durable power of attorney and cannot express his will and make decisions, as he is unable to communicate (totally unresponsive, unconscious, etc.), family members can be consulted about the patient’s presumed wishes regarding invasive treatments. This is provided they are available – recognizing that locating them in times of emergency and scarcity is not easy.
31. Despite the desire to ascertain the patient’s wishes grounded in the respect for personal autonomy, one must be aware that if the scenario is realized wherein the treatment of all patients is impossible, the medical staff may decide not to treat a particular patient. Failure to provide treatment may be in accordance with the patient’s original request, or contrary to the patient’s original request. In other words, in a time of resource scarcity, concurrence will occur only for the wishes of those patients who asked to avoid invasive treatment. Therefore, there are those who believe that in a state of scarcity there is no point in ascertaining the wishes of patients because under these circumstances the patients’ desires will not be considered, so that ascertaining their wishes is misleading. Conversely, it can be argued that there is inherent value in ascertaining a person’s wishes and that in those cases where the individual’s treatment preference coincides with the therapeutic decision, this knowledge provides relief for both the patient and the medical staff.
32. The senior physician or the triage committee representative should inform the patient who can express his wishes, or the relatives of the patient who cannot express his wishes, of any adverse triage decision made for him. The family, however, has no right to oppose such decisions.

## Chapter 3: Applied Guidelines

### A. In the Current Emergency with No Scarcity in Israel

1. In the present situation – at the time of publication of this document – the State of Israel is not in a state of insufficient life-saving resources or skilled manpower. Therefore, the conditions for applying the triage guidelines of this position paper do not apply. Rather, applicable at present are the usual rules and principles pertaining to times when there are no shortages of life-saving resources or skilled manpower.

### B. In an Emergency with Scarcities

2. If and when – and with prayer and hope that it does not occur – the State of Israel’s healthcare system reaches insufficiency regarding life-saving resources or skilled manpower, despite all feasible efforts to expand reserves or obtain additional resources, and after the Ministry of Health has declared a transition to an emergency state due to shortage conditions, the triage guidelines outlined in this position paper will apply.

### C. The Will of the Patient

3. A patient who can express his will: The attending physician, whether for a COVID-19 patient or a non-COVID-19 patient, must ascertain whether the patient wrote an advance medical directive or appointed a durable power of attorney for medical decisions. If the answer is no, the doctor should explore with the patient whether the patient wants to clarify his treatment preferences should his condition deteriorate. A patient who is not interested in discussing this issue should have his wishes respected, but if he is interested in expressing his desires, a conversation should be conducted. This should be done with the requisite sensitivity to the situation and its inherent limitations (fears and anxieties, difficulties of understanding and awareness, personal protective equipment worn by the physician encumbering regular interaction and the limitations of contact through telephone or video). It is important to inform the patient about the chances of success of invasive treatment, if he comes to need it, and to note the various alternatives including prevention of suffering by providing palliative care. The patient’s wishes must be respected as much as possible under the circumstances, whether they involve avoidance of certain treatments or the demand for treatment. The conversation and conclusions should be documented.
  At the stage of deterioration – as long as the patient is capable of having a discussion, it is appropriate to reevaluate and determine his wishes in relation to the treatments required in the new situation and to present the various alternatives as mentioned above. This must be done with great sensitivity and consideration of the physical and mental state of the patient and his cultural values. If the patient does not wish to express his wishes at this stage, it should be determined whom he wants to make decisions for him.
4. A patient who cannot express his will as he is unable to communicate (totally unresponsive, unconscious, etc.): It is necessary to check for the existence of a written advance medical directive, a durable power of attorney, a guardian or a documented summary in the medical record exist, and if found then to act accordingly. In the absence of such information, it is necessary to ascertain with family members whether the patient’s position regarding invasive treatments in severe medical conditions is known. The wishes of family members are not in themselves an acceptable consideration in this regard.

### D. Medical Triage Rules

5. The Medical Subcommittee comprised 8 senior and experienced physicians from the following medical disciplines: Intensive Care, Internal Medicine, Geriatrics, Medical Administration, Trauma, Psychiatry and Palliative Medicine.
6. The medical principles and criteria for triage admissions to the ICU and for ventilation were established only for situations with a scarcity of life-saving resources.
7. The principal starting points of the supreme value of life and the basic equality of all human beings remain in force even in emergencies with a scarcity of life-saving resources.
8. Indeed, when there is scarcity of life-saving resources and when many people need ICU beds, respirators or ECMO, there is no choice but to triage the patients who will benefit from these measures.
9. The principle of triage decisions is only on a medical basis regarding the success of treatment with greater chances of survival and of weaning from the ventilator. The goal in extreme conditions is to save as many people as possible without any other considerations that differentiate people since all people are equal and the value of their lives is equal. Under no circumstances should physiological triage in extreme situations be interpreted as a judgment on the value of any person’s life.
10. The triage decisions will only take place on a medical basis as described above. Therefore, the following factors will not be included in the triage decisions:
  - Religion, race, gender, nationality, country of origin, sexual orientation, socioeconomic status, social status, family status, citizenship, occupation, etc.
  - Years of life including age considerations: The chronological age itself is not a legitimate consideration in triaging life-saving treatment but only as a part of the combination of risk factors.
  - Disability: Disability in itself is not a legitimate consideration in triaging life-saving treatment but only as part of the combination of risk factors.
  - Circumstances that may be considered to be the patient’s fault, including negligence which may have caused a COVID-19 infection.
  - Merits to the patient from his past actions to this time.
11. Healthcare professionals, even if infected while treating COVID-19 patients, will not be given priority unless it is necessary to overcome staff shortages, either by facilitating return to work after their recovery or as an incentive to volunteering. When there is medical equality between two patients, healthcare professionals will receive priority.
12. These rules will apply in the same way to all workers who in their job come into contact with COVID-19 patients.
13. These medical triage methods are intended to be used by ICU specialists, as well as by physicians who will treat ventilated patients outside ICUs, who are often inexperienced making decisions in extreme situations.
14. The triage framework must address not only COVID-19 patients but all patients in need of life-saving treatments. Therefore, COVID-19 patients should be in a pool with other patients who require life-saving treatments for any medical condition. Patients with other illnesses should not be deprived of hospitalization and necessary treatment for the benefit of COVID-19 patients.

### E. Medical Assessment Tools for Triage

**Figure 1:**
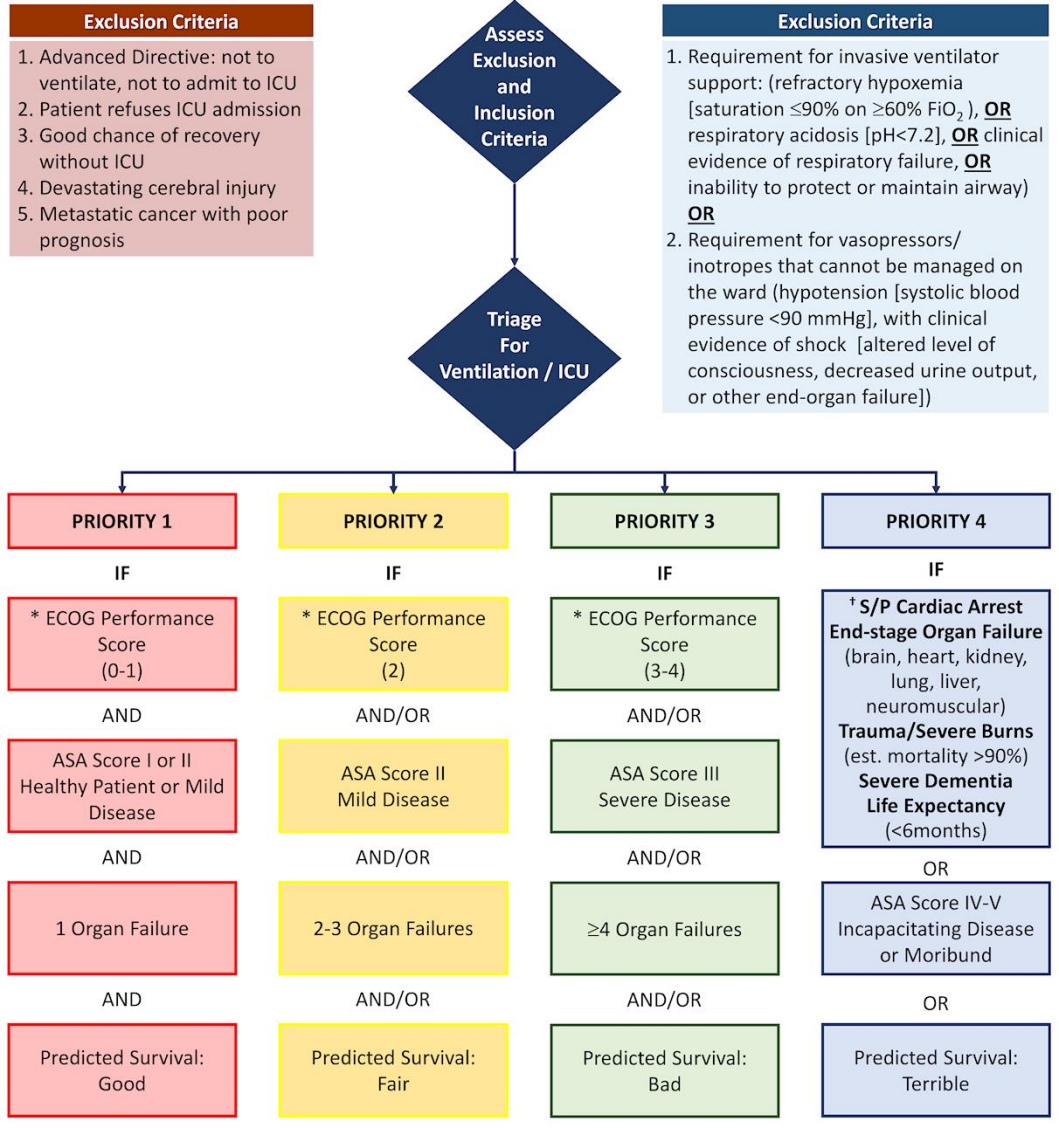
Flowchart and Priority Tables for Emergencies with Limited Life-saving Therapies *The degree of functioning should only be considered for short-term survival, especially for the disabled. † An acute cardiac arrest or with significant brain damage. Tie-breaking: Triage is based on saving the most lives considering acute and chronic illnesses. If there is still a tie, proceed based on medical considerations – use first come, first served. ASA, American Society of Anesthesiologists; FiO_2_, fraction of inspired oxygen; ICU, intensive care unit; ECOG, Eastern Cooperative Oncology Group Legend continued on the following page. Adapted from Figure 1 of Sprung et al.^2^ Used with permission. The Creative Commons license does not apply to this content. Use of the material in any format is prohibited without written permission from the publisher, Wolters Kluwer Health, Inc. Please contact permissions@lww.com for further information. The triage decision is a complex clinical determination made when ventilators or ICU beds are limited and are not sufficient for all patients who need them. Structured decision-making is important to maximize transparency and improve consistency in decision-making. To do this, it is essential to assess the expected benefit to the patient and to compare it to the expected benefit to another patient (for example, comparing outcomes of ventilation or ICU admission versus the expected outcome if the patient remains on the ward or without ventilation). This applied algorithm outlines a recommended process for individual triage measurements without any reference to group affiliation as a valid medical tool.The physician should refer to all the measurements, especially at the ECOG Performance Score solely based on the relevant medical assessment for the success of treatment and the likelihood of survival and weaning from the ventilator. No other consideration should be taken into account that distinguishes between individuals, such as age itself, disability per se, or disease per se, but only insofar as the variable medically predicts chances of survival. It must be remembered that all human beings are equal and their value of life is equal. Under no circumstances should medical triage decisions in extreme situations be interpreted as a judgment on the value of a person’s life.The triage process begins with exclusion criteria:The initial exclusion criteria are based on exclusion criteria used under ‘normal’ conditions.If no exclusion criteria are met, patients must meet one of the inclusion criteria which means their condition is severe enough to require intensive care or ventilation.Once a patient does not meet the exclusion criteria and meets the inclusion criteria, he is eligible to be admitted to the ICU or connected to a ventilator.The criteria for a ventilator or ICU admission are next individually inspected according to the priorities in the flow chart and ranking tables.Prioritization is based on priority ranking from 1 to 4 (Priority 1 followed by Priority 2 followed by priority 3 and finally Priority 4).The selected triage tools are multidimensional. They include Performance Score (ECOG), Comorbidities (ASA), organ system failure and an overall assessment of the short-term survival chances. The medical literature shows that using a number of different tools increases the accuracy of predictability compared to using a single tool. In addition, the selected measurements are those that enable rapid assessment, which is essential in emergency situations. Re-assess priority every 24h for patients waiting for ventilation or ICU admission. Re-assess ICU patients at day 10–14 or in the event of significant worsening of the patient’s condition, consideration should be given to transferring the patient to a regular ward or restrict treatment.

**Figure 2:**
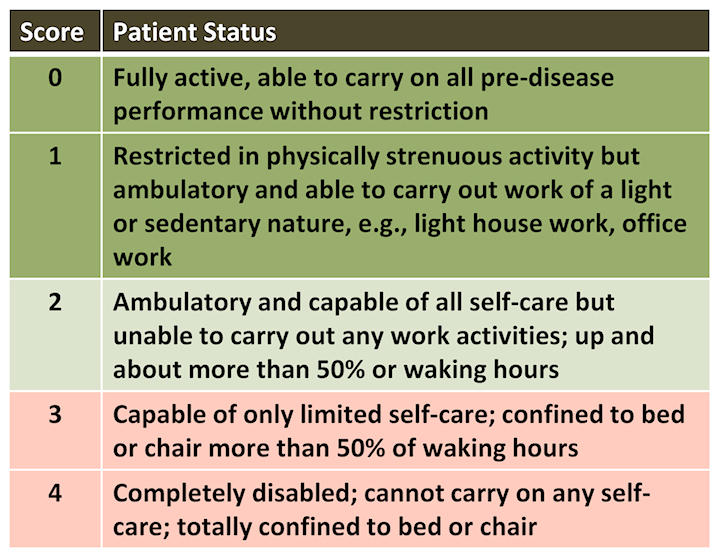
The Eastern Cooperative Oncology Group (ECOG) Performance Score Adapted from the ECOG Performance Status.^3^

**Figure 3:**
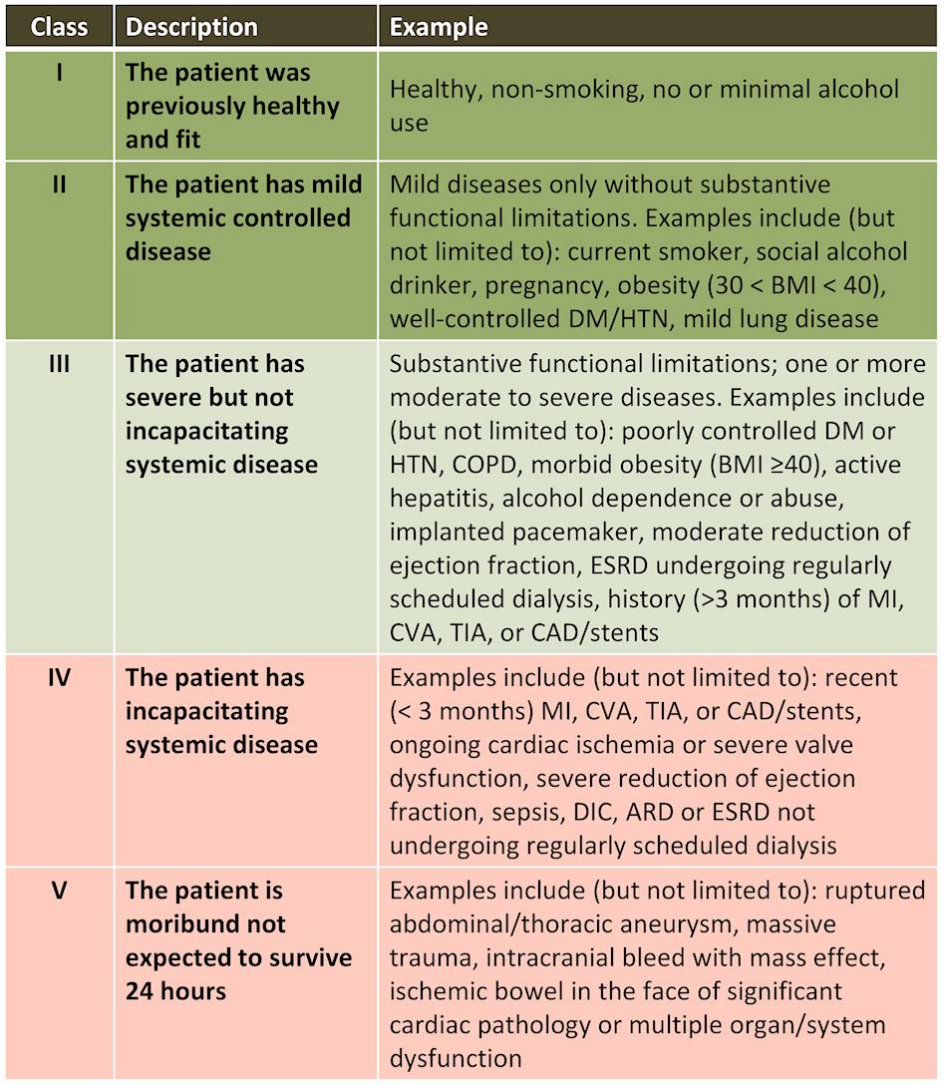
The American Society of Anesthesiologists (ASA) Score ARD, acute respiratory distress; BMI, body mass index; CAD, coronary artery disease; COPD, chronic obstructive pulmonary disease; CVA, cerebrovascular accident; DIC, disseminated intravascular coagulation; DM, diabetes mellitus; ESRD, end-stage renal disease; HTN, hypertension; MI, myocardial infarction; TIA, transient ischemic attack. Adapted from the ASA Physical Status Classification System.^4^

15. In order to provide the physician with efficiency in making triage decisions in emergency situations under extreme pressure, the assessment tool should meet the following requirements:
  - It should contain valid measurements, each of which – according to the medical literature – predicts survivability.
  - It should be complete and concise on a single page, so as to assist physicians in making quick decisions when facing numerous severely ill patients and limited life-saving resources.
  - It should be simple, easy to understand and use, enabling quick assessments.
  - The measurements should be listed according to the amount of time required to evaluate them, with the quickest preceding the more time-consuming and with logical progression, so that if the criteria are not met, the physician can quickly move on to the next candidate. This will serve to optimize the triage process, which is expected to occur under conditions of time pressure and limited medical staff.
16. In order to triage on a medical basis the medical subcommittee evaluated a number of familiar tools for intensive care and ventilator triage during epidemics. The members of the Commission discussed at length various proposals of algorithms.
17. In general, the medical subcommittee thought that assessment tools should be multidimensional rather than based solely on a particular measurement, because multidimensional assessments have been demonstrated to yield better predictability of short-term survival and of weaning from ventilation.
18. The several measurements that predict short-term survival and weaning from ventilation are: background diseases, functioning of the vital organ systems and functional ability.
19. The commission was aware of the social implications of using a functional measure, especially for people with disabilities. As noted above, disability by itself should not be considered as a criterion for triage decisions, as there are many disability situations that have no bearing on short-term survival. However, there are functional deficiencies that are relevant to short-term survival and are only reflected in the evaluation of performance measures, and not in system failure or background diseases, such as functional deficits that affect lung function. Therefore, it is also emphasized in the applied guidelines for physicians that the use of the function index is individual and the degree of function should only be assessed in relation to the chances for short-term survival.
20. The triage criteria according to functional scores relate to all groups, pertaining to both intra-group and inter-group comparisons, and are determined only on the basis of individual indices. Most people who will be assigned functional scores are not people with disabilities. There is, however, a particular sensitivity in defining functioning for people with disabilities. Therefore, it should be emphasized that when evaluating the functional classification, only the functional aspects related to survivability should be considered.
21. In general, the medical rationale for using physical function classifications is based on the medical literature indicating that the lower the daily function, the lower the muscular activity and respiratory function and the likelihood of survival also decreases. Specifically, the attending physician must address the degree of functioning in relation to the likelihood of survival. For example, a Paralympic athlete with amputated legs is likely to have better survival chances than someone suffering from heart failure, even if the athlete needs help with some day-to-day activities.
22. The commission believes that a functional measure cannot be excluded as it is an independent predictor of survival. Therefore, its exclusion would diminish the accuracy of the triage process and may result in incorrect and medically discriminatory prioritizations. In particular, there are certainly cases where there will be equal scores for disease / organ function, with only the assessment of functional ability allowing the choice of a patient with a higher chance of survival.
  Some examples include: two patients on chronic dialysis for renal failure whose comorbidities and organ failures are equal, but whose survival is likely to be very different if one is active while the other is bedridden; this information is obtained only from a performance score. Similarly, the chances of survival of an amputee in a good functional state compared to a bedridden amputee. Likewise, the chances of survival of an amputee in a good functional state are equal to a non-amputee in the same functional condition, and both have much higher survival chances than an elderly patient in a low-functioning condition.
23. Candidate medical assessment tools considered for assessing function included:
  - Clinical Frailty Scale (CFS) – This measure was originally developed to assess the functioning of the elderly for life expectancy assessments. It contains the following variables: weakness, exhaustion, weight loss, low physical activity, disturbed equilibrium, decreased walking pace, visual impairment and cognitive disorder. This scale contains 9 variables so it is less effective for a quick assessment in an emergency where immediate decisions are required.
  - Activities of Daily Living (ADL) – This is an accepted measure of function evaluation, also originally intended to assess the functioning of the elderly in order to assess life expectancy. The measurement includes routine tasks that a person performs during his daily routine including basic feeding, bathing, movement (transferring and getting out of bed), sphincter control and using the bathroom. The ADL questionnaire is relatively long, requires counting points and calculating a final score. It is therefore less efficient in an emergency where quick decisions are needed.
24. In light of all the considerations presented above, the Commission decided to use 4 multidimensional triage tools: Performance Score (ECOG), Comorbidities (ASA), Organ System Failure and an overall assessment of the short-term survival prospects. As noted above, the medical literature indicates that using several different tools increases the accuracy of prediction compared to using a single tool. In addition, the selected measurements are those that enable rapid assessment which is essential in emergency situations.
25. The following are the chosen evaluation tools:
  - ECOG Performance Score – This measure was developed some 35 years ago to predict the survival of cancer patients. Since then, this measure has been found to be a significant predictor of survival in many medical conditions including the elderly, patients with pneumonia and ICU patients. The measure has 5 performance levels based on variables that are easy to assess in a short time. Therefore, it was selected as the functional measurement appropriate for use in emergencies.
  - B. Comorbidities – For the assessment of comorbidities, the American Society of Anesthesiologists (ASA) Score was chosen. It is well known, contains concise descriptions, and is logically organized. This is instead of displaying a long and detailed list of background diseases. In order to identify Priority 4 patients, we chose to use general diagnoses, rather than employing a detailed list of specific diagnoses. A long list of diagnoses makes it difficult to make a quick assessment, while in any case such a list is never exhaustive.
  - C. Organ System Failure – The approach chosen to determine Organ System Failure (OSF) was the number of systems that failed rather than the Sequential Organ Failure Assessment (SOFA) score which requires scoring and additional calculations. The SOFA score was originally developed for patients with organ failure from sepsis, evaluating ICU patients according to the failure of one or more vital organ systems. This score was not originally intended for triage during a shortage and only refers to the degree of systemic failure, with the aim of assessing illness severity and ICU mortality. It should also be noted that in the past, the SOFA score was not found useful when examined retrospectively in epidemic situations. In epidemics, it was found that most patients who, according to the SOFA score, would not be admitted to the ICU, actually survived. Moreover, specifically regarding the COVID-19 pandemic, most of these hospitalized patients suffer from only one organ system failure, which significantly reduces the ability to triage based on the SOFA score.
26. This integrated assessment tool was developed by an international group of ICU triage experts drawing on their experience from previous epidemics and with the help of data obtained from clinicians dealing with severe resource shortages during the current COVID-19 pandemic. Practical applications of this assessment tool and use in practice during the current pandemic have been a major consideration in its construction.
27. The Chair of the Medical Subcommittee, Professor Charles Sprung, was actively involved in developing this algorithm with other professional committees in Israel and around the world. The Israel Society of Critical Care Medicine has adopted the current algorithm for ICUs throughout Israel.
28. The current algorithm (Figure 1),^2^ the Eastern Cooperative Oncology Group (ECOG) performance score (Figure 2),^3^ and the American Society of Anesthesiologists (ASA) scores (Figure 3),^4^ were adapted from the algorithm published in Critical Care Medicine.^2^
  The authors of this publication are world-renowned experts in their medical field and members of well-known professional international groups. They come from Israel, the USA, the UK, Spain and China. Some of the authors have personally experienced triage decisions both in regular times and in pandemics, including the current COVID-19 pandemic.
29. This tool provides a component of objectivity with the aim of helping to maintain a systematic determination of the short-term survival chances. However, it is not a substitute for triage judgment by an experienced intensive care clinician.
30. This assessment tool outlines a recommended process for obtaining individual triage measurements, without any reference to group affiliation.
31. In general, the medical rationale for using degrees of physical function is based on the medical literature that indicates that the lower the daily functioning due to weakness or fragility, the lower the muscular activity and respiratory function and hence the likelihood of survival decreases. Specifically, the attending physician must address the degree of functioning in relation to survival.
32. Each decision utilizes agreed-upon criteria for ventilation and intensive care at a specific time and depends on the resources available and the number of patients awaiting the scarce resource. (For example, a more stringent threshold may be needed during the pandemic’s peak and a less stringent threshold at the beginning and towards the end of the pandemic).

### F. Palliative Care

33. Even in emergencies and resource scarcity in which a triage process occurs, when a decision is made not to admit the patient into the ICU, not to mechanically ventilate him, or to discharge him from the ICU, the patient must continue to receive palliative care according to accepted medical standards, and he should not be neglected physically nor mentally. The Ministry of Health should establish procedures to ensure optimal palliative care, whether as part of existing COVID-19 wards or by assigning palliative COVID-19 wards.
  In addition, the Commission recommends that the Ministry of Health examine the advantages and disadvantages of establishing a special COVID-19 hospice subject to the conditions of the previous section with the aim of maximizing palliative care.
34. It is desirable that the severely ill and especially dying patients, shall have the opportunity to say farewell to a close person in a humane fashion including a visit with direct contact. However, it is important to reduce the risk that such contact will infect the visitor and consequently produce a secondary infectious chain. Therefore, it is recommended that the physical farewell be limited to one related person. Whenever possible, a relative who was infected with COVID-19 and has recovered should be preferred, especially if serological examinations – insofar as they are available and reliable – prove him to be immune. However, if there is another family preference or in the absence of such a relative, the direct visit of one relative should be allowed on the condition that he be fully protected, will agree to take the risk, and after the visit will commit to follow the medical instructions recommended (for example, to go into quarantine or undergo testing). In the same way, the physical presence of a member of the clergy should be allowed for patients who so desire.
35. Physicians and staff involved in triage decision-making should be given as much ethical assistance as needed, as well as the necessary support and emotional assistance, given the challenging nature of the requisite decisions.

### G. Transparency

36. The medical team must do as much as it can to document its decisions and clearly state the assessment of the patient’s chances of survival as well as the decision to provide or not to provide the therapeutic resource.
37. Patients or their families should be presented early with the treatment plan appropriate for the patient’s condition in accordance with the triage criteria and with the principles and benefits of palliative care, thus avoiding an impression that if it is decided not to prioritize the patient, he will be neglected and will not receive supportive care.

### H. Institutional and National Prioritization Committees

38. Every medical institution will appoint a special ad hoc institutional triage committee to be available to the senior medical specialist for consultation for any case he finds appropriate.
39. This triage committee will consist of 4 people: a senior medical specialist who is not treating COVID-19 patients, a senior nurse, an ethicist, and a member of the clergy. When an urgent decision is needed – two of the four members of the COVID-19 committee are sufficient.
40. This committee can also assist the senior physician in explaining the triage decisions to the patient’s family.
41. This committee has consultative authority but any triage for each individual patient will be made by a senior intensive care specialist after consulting another medical specialist. In the absence of a senior intensive care specialist, by two senior physicians based on the criteria in this position paper.
42. The Ministry of Health will appoint a National triage committee to discuss key issues that arise during the epidemic. Its members will be: Senior Specialist in Intensive Care Medicine, Internal Medicine or Geriatrics Specialist, Senior Nurse, Lawyer, Ethicist, Member of the Clergy, and Community Representative.

## Abbreviations

COVID-19: coronavirus disease 2019
ICUs: intensive care units
OSF: organ system failure
SARS-CoV-2: coronavirus infections
SOFA: sequential organ system failure assessment.

## Appendix 1 Commission Members

### Co-chairs of the Commission

- Rabbi Prof. Avraham Steinberg – Co-Chair of the National Bioethics Council
- Prof. Efrat Levi-Lahad – Co-Chair of the National Bioethics Council
- Dr. Tami Karni – Chair of the Ethics Bureau of the Israel Medical Association

Assistants: Ms. Nurit Desau – Research Assistant to the National Bioethics Council; Adv. Rafi Twizer – Health Ministry Coordinator; Ms. Deganit Lahav – Administrative Assistant to the National Bioethics Council.

### Medical Subcommittee

- Prof. Charles Sprung, Chairman – Director Emeritus, General Intensive Care Unit, Department of Anesthesiology, Intensive Care and Pain, Hadassah Medical Center, Jerusalem
- Prof. Jonathan Halevy, Member – former CEO of Shaare Zedek Medical Center, Jerusalem
- Prof. Yaron Niv, Member – Senior VP for Quality and Safety, Head of Ventilation Action Team for COVID-19 Patients in hospitals, Ministry of Health
- Prof. Ofer Merin, Member – CEO of Shaare Zedek Medical Center, Jerusalem
- Prof. Pesach Schwartzman, Member – Chairman of the Israeli Association for Palliative Medicine
- Prof. Moshe Sonnenblick, Member – Director of Clinical Geriatric Unit, Shaare Zedek Medical Center, Jerusalem
- Dr. Adi Nimrod, Member – Director, General Intensive Care Unit, Ichilov Medical Center, Tel Aviv
- Prof. Israel Strouss, Member – Director of Psychiatry, Mayanei HaYeshua Medical Center, Bnei Brak

### Philosophical-Ethical-Social Subcommittee

- Prof. Noam Zohar, Chairman – Director, Graduate Program in Bioethics, Department of Philosophy, Bar Ilan University, Ramat Gan
- Prof. David Heyd, Member – Department of Philosophy, The Hebrew University, Jerusalem
- Prof. Yael HaShiloni-Dolev, Member – Department of Sociology and Anthropology, Ben-Gurion University, Beer Sheva
- Prof. Ruth Landau, Member – School of Social Work and Social Welfare, The Hebrew University, Jerusalem
- Dr. Efrat Ram-Tiktin, Member – Department of Philosophy, Bar Ilan University, Ramat Gan

### Legal Subcommittee

- Dr. Gil Siegal, Chairman – Director, Center for Health Law, Bioethics, and Health Policy, Law Faculty, Ono Academic College
- Judge Salim Jubran, Member – Deputy Chief Justice, Supreme Court, emeritus
- Prof. Shai Lavi, Member – Head of the Van Leer Institute, Jerusalem
- Attorney Talia Agmon, Member – Deputy Chief Legal Counsel, Ministry of Health

### Halachic/Religious Subcommittee

- Rabbi Prof. Avraham Steinberg, Chairman – Co-Chair of the National Bioethics Council
- Rabbi Brigadier General Eyal Krim, Member – Chief Military Rabbi, Israel Defense Forces
- Rabbi Prof. Yigal Shafran, Member – Head of the Department of Medicine and Halacha, Chief Rabbinate of Jerusalem
- Rabbi Dr. Mordechai Halperin, Member – Director of the Schlesinger Institute for Medical Halachah Research, at the Shaare Zedek Medical Center, Jerusalem
- Rabbi Avraham Manela, Member – CEO of chevrah kadishah (Burial society), Tel Aviv
- Qadi Muhammad Abu-Abeid, Member – Judge of the Sharia Court of Appeal (Islam)
- Dr. Etienne Lepicard, Member – Center for Medical Education, Hadassah and the Hebrew University, Jerusalem and Director of “Bet Hagat,” the home for interfaith-artistic encounters in Jerusalem (Christianity)
